# Berberine Prevents and Reverses Gastric Spasmolytic Polypeptide-Expressing Metaplasia by Modulating M2 Macrophage Polarization

**DOI:** 10.3390/ijms27198528

**Published:** 2026-09-24

**Authors:** Jia-Le Li, Shi-Yi Ge, Xing-Xing Zhou, Rong Yan, Ke-Xin Lin, Si-Ran Wu, Xiao-Dong Qu, Na Wang, Jian Wu, Yong-Quan Shi

**Affiliations:** State Key Laboratory of Holistic Integrative Management of Gastrointestinal Cancers, National Clinical Research Center for Digestive Diseases, Department of Gastroenterology & Hepatology, Xijing Hospital, The Fourth Military Medical University, Xi’an 710032, China; lijiale0404@163.com (J.-L.L.); geshiyi0730@163.com (S.-Y.G.); 15393068898@163.com (X.-X.Z.); yanrong0310@126.com (R.Y.); linkexin505@163.com (K.-X.L.); 18402950284@163.com (S.-R.W.); quxd052733@163.com (X.-D.Q.); 15289387077@163.com (N.W.)

**Keywords:** berberine, SPEM, tamoxifen, M2 macrophage, S100A6

## Abstract

Spasmolytic polypeptide-expressing metaplasia (SPEM) is a critical gastric precancerous lesion with no approved pharmacological intervention. Berberine (BBR), a natural isoquinoline alkaloid, exhibits pleiotropic anti-inflammatory and antitumor activities. This study investigated whether berberine prevents and/or reverses tamoxifen-induced SPEM in mice and elucidated the underlying mechanisms. A tamoxifen-induced SPEM mouse model was employed with berberine administered via oral gavage in both preventive and therapeutic regimens. Gastric mucosal pathology was assessed by H&E staining, immunohistochemistry, and immunofluorescence. Transcriptomic profiling, bioinformatic analyses, and single-cell RNA sequencing data integration were performed to identify key molecular targets. Berberine significantly attenuated tamoxifen-induced gastric glandular atrophy, accelerated gland recovery, and reduced the number of TFF2^+^ and AQP5^+^ SPEM cells. Transcriptomic analysis revealed that berberine reversed the tamoxifen-induced upregulation of JAK-STAT and NF-κB signaling pathways. Consistently, berberine suppressed CD163^+^ M2 macrophage infiltration into the gastric mucosa in a time-dependent manner. Integrative single-cell analysis identified that S100A6 expression progressively increased with malignant progression. S100A6 was also elevated in SPEM and downregulated upon berberine treatment, which might be associated with M2 macrophage infiltration. Berberine prevents and reverses gastric SPEM, at least in part, by inhibiting M2 macrophage polarization and infiltration, possibly via regulating the JAK-STAT/NF-κB pathway and S100A6 expression. These findings provide preclinical evidence supporting berberine as a candidate therapeutic agent for SPEM.

## 1. Introduction

Gastric cancer (GC) remains one of the leading causes of cancer-related mortality worldwide, with most patients diagnosed at an advanced stage when curative therapies are limited [1,2]. More than 80% of GCs are the intestinal-type GC, and their development follows the classic Correa’s cascade: chronic superficial gastritis → chronic atrophic gastritis → intestinal metaplasia → dysplasia → carcinoma [3,4]. In recent years, spasmolytic polypeptide-expressing metaplasia (SPEM) has been recognized as a second, likely earlier, metaplastic pathway linked to intestinal-type gastric carcinogenesis [5].

Cumulative evidence suggests that TFF2^+^/AQP5^+^ SPEM cells originate from the transdifferentiation of gastric chief cells [6,7]. Under acute injury, SPEM contributes to mucosal repair. However, under persistent inflammatory stimulation, it may initiate the progression of precancerous lesions [7,8]. Mechanistically, M2 macrophage polarization and infiltration, orchestrated by the IL-33-ST2-IL-13 signaling axis, play a pivotal role in SPEM pathogenesis [9,10]. Depletion of macrophages abrogates SPEM development in mice, highlighting the significance of M2 macrophages in SPEM pathogenesis [9]. Clinically, SPEM is detected in mucosa adjacent to early gastric cancer and in remnant gastric mucosa after endoscopic resection, at rates significantly higher than those of intestinal metaplasia [11,12,13]. Notably, SPEM cells have been identified at the base of incomplete intestinal metaplasia, which carries the highest malignant risk [14,15]. Crucially, unlike intestinal metaplasia, which is largely refractory to reversal [16], SPEM can regress [17,18]. Nevertheless, no pharmacological agent is currently approved to prevent or reverse SPEM.

Berberine, an isoquinoline alkaloid from Coptis chinensis and Phellodendron amurense, exhibits multimodal pharmacological activities including anti-inflammatory, antimicrobial, and antitumor effects [19,20,21]. As reported, berberine could exert multi-step therapeutic effects on the Correa’s cascade. Firstly, berberine attenuates chronic atrophic gastritis by suppressing the IRF8/IFN-γ signaling axis [22]. Secondly, berberine reverses intestinal metaplasia and dysplasia by modulating the cell cycle, autophagy, and apoptosis [23,24,25]. Lastly, berberine inhibits the proliferation and invasion of GC cells and induces apoptosis and autophagy in GC cells [26,27]. Despite its multifaceted therapeutic effects, whether berberine exerts any effect on SPEM remains unknown.

Here, we tested the hypothesis that berberine could prevent and/or reverse tamoxifen-induced SPEM in mice. Using histopathological, transcriptomic, and integrative single-cell analyses, we demonstrated that berberine hydrochloride attenuated SPEM, possibly by inhibiting JAK-STAT/NF-κB and S100A6-dependent M2 macrophage polarization and infiltration. Our findings, for the first time, demonstrate the preventive and therapeutic effects of berberine on SPEM, thereby offering a potential therapeutic strategy for clinical reversal of SPEM and contributing to the development of comprehensive approaches for the prevention of GC.

## 2. Results

### 2.1. High-Dose Tamoxifen Successfully Induced SPEM

The model group and the control group were treated with tamoxifen and tamoxifen solvent by intraperitoneal injection for 3 days, respectively (Figure 1A). H&E staining revealed that compared with vehicle-treated controls, mice receiving tamoxifen (250 mg/kg, i.p., 3 days) exhibited marked gastric glandular disorganization, loss of normal straight tubular architecture, glandular tortuosity, dilatation and cyst formation, and a significant reduction in gland height, along with a substantial decrease in eosinophilic parietal cells in the mid-gland region (Figure 1B,F). Immunohistochemical and immunofluorescence staining further showed that the number of ATP4α^+^ parietal cells was significantly reduced, while TFF2^+^ and AQP5^+^ cells at the gland base were markedly increased (Figure 1C–E,G–K). All these results confirm successful SPEM induction by high-dose tamoxifen.

### 2.2. Prophylactic Berberine Attenuates SPEM

Berberine pre-treatment (100 mg/kg, p.o., 10 days, starting 7 days before tamoxifen) did not fully restore normal glandular architecture or reverse glandular disorganization induced by tamoxifen, as revealed by H&E staining, but significantly increased gland height and notably preserved parietal cell numbers in the mid-gland region compared with vehicle-treated SPEM controls (Figure 2B,C,E–G,K). Immunohistochemical and immunofluorescence staining further confirmed that ATP4α^+^ parietal cells were significantly increased in the prevention group (Figure 2C,E,G,K). Additionally, the number of AQP5^+^ cells at the gland base was significantly reduced (Figure 2D,I), whereas TFF2^+^ cells showed a decreasing trend that did not reach statistical significance (Figure 2D,E,H,J). These findings indicated that berberine partially prevented tamoxifen-induced SPEM by attenuating parietal cell loss.

### 2.3. Therapeutic Berberine Accelerates SPEM Resolution

Berberine administered after SPEM induction significantly accelerated glandular recovery. Following tamoxifen-induced SPEM, mice received daily oral gavage of berberine or vehicle for 14 consecutive days and were sacrificed at days 3, 7, 10, and 14 after treatment initiation for gastric tissue collection (Figure 3A). After 3, 7, and 10 days of treatment, gland height was significantly greater in berberine-treated mice than in time-matched vehicle controls. Notably, gland height in the treatment group at day 10 had already matched that of vehicle controls at day 14, reflecting an accelerated recovery trajectory (Figure 3B,D). The number of ATP4α^+^ parietal cells increased significantly, and the number of TFF2^+^ and AQP5^+^ SPEM cells at the gland base decreased significantly at days 3–10 in berberine-treated mice compared with time-matched controls. Furthermore, ATP4α^+^ parietal cell number in berberine-treated mice at day 7 had already reached the level of vehicle controls at day 10, and by day 10 it matched that of controls at day 14; a similar accelerated pattern was observed for TFF2^+^ and AQP5^+^ cell reduction, indicating faster SPEM resolution in the berberine group (Figure 3C,E–L). Thus, these results indicate that berberine promotes glandular recovery and alleviates tamoxifen-induced SPEM.

### 2.4. Transcriptomic Profiling Reveals Immune Pathways Modulated by Berberine

RNA-seq identified 4290 DEGs between the SPEM model and control groups (2224 upregulated, 2066 downregulated), as well as 106 DEGs between the prevention group and SPEM model group (71 upregulated, 35 downregulated). In the treatment group, 3723 (1842 upregulated, 1881 downregulated), 442 (393 upregulated, 49 downregulated), 1257 (604 upregulated, 653 downregulated), and 1445 (1047 upregulated, 396 downregulated) DEGs were identified at 3, 7, 10, and 14 days, respectively (Figure 4A,B).

In the model group, the up-regulated genes mainly participated in biological processes such as cell process regulation, immune system process regulation, and inflammatory response, and were related to the cell cycle, NF-κB signaling pathway, and JAK-STAT signaling pathway, etc., while the down-regulated genes were mainly enriched in processes such as cell structure and cell development, as well as oxidative phosphorylation and protein degradation, etc. The up-regulated genes in the treatment group after 3 days mainly participated in lipid metabolism, cell structure, protein binding, oxidative phosphorylation, protein metabolism, and heat production, etc., while the down-regulated genes were mainly enriched in biological regulation, signal transduction, protein binding, etc., and were related to cytokine interaction, JAK-STAT signaling pathway, NF-κB signaling pathway, and TNF signaling pathway, etc. The up-regulated genes in the treatment group after 7 days mainly participated in lipid metabolism, cell structure, protein binding, oxidative phosphorylation, protein metabolism, and heat production, etc., while the down-regulated genes were mainly enriched in biological regulation, signal transduction, protein binding, etc., and were related to cytokine interaction, PPAR signaling pathway, Calcium signaling pathway, and ECM-receptor interaction, etc.

Consistent with previous reports and our immunohistochemical and immunofluorescence findings, SPEM in the mouse gastric mucosa can spontaneously resolve by 14 days after SPEM establishment; accordingly, the therapeutic effects of berberine on SPEM mainly manifested at the first three time points. The intersection of model-upregulated genes with berberine-downregulated genes at days 3 and 7 yielded 64 common DEGs (Figure 5C), including the known SPEM marker *Wdfc2*, which was upregulated in the tamoxifen-induced SPEM model and significantly downregulated after berberine treatment, consistent with literature reports. Further GO analysis indicated enrichment in cell differentiation, cell process regulation, stimulus regulation, and immune system regulation (Figure 5D). KEGG analysis highlighted JAK-STAT, NF-κB, PI3K-AKT, and TNF signaling pathways, etc. (Figure 5D). GSEA confirmed that both JAK-STAT signaling and NF-κB signaling were significantly upregulated in the SPEM model and significantly downregulated after 3 days of berberine treatment. These results suggest that berberine may exert alleviating effects on SPEM by regulating these pathways (Figure 4E).

### 2.5. Berberine Suppresses M2 Macrophage Polarization and Infiltration

As demonstrated in the previous section, JAK-STAT and NF-κB signaling pathways were significantly upregulated in the model group and significantly downregulated in the berberine treatment group after 3 days of intervention. Given the established role of JAK-STAT and NF-κB signaling in M2 macrophage polarization and tissue infiltration, we further examined macrophage-related transcripts. The M2 macrophage marker *CD163* was markedly upregulated in the SPEM model and decreased progressively in a time-dependent manner after 3, 7, and 10 days of berberine treatment (Figure 4A,C). Similar change patterns were observed for the M2-associated genes *Lyve1* and *Cd9*. CD163 immunohistochemistry confirmed these findings at the tissue level. In the SPEM model, dense focal infiltrates of CD163^+^ M2 macrophages were observed in the gastric mucosal lamina propria and intergranular space. Berberine treatment significantly reduced the number of CD163^+^ cells at all three time points, with the degree of reduction becoming more prominent as treatment duration increased (Figure 5B,D). These results indicated that berberine suppressed M2 macrophage polarization and gastric infiltration, likely by inhibiting the JAK-STAT and NF-κB pathways, thereby exerting therapeutic effects on gastric mucosal lesions.

### 2.6. S100A6 Could Be the Target Molecule of Berberine

In order to identify key molecules that may contribute to the progression of gastric precancerous lesions and respond to berberine, we performed a systematic bioinformatic analysis of public single-cell RNA sequencing (scRNA-seq) data and our transcriptomic data. Public scRNA-seq datasets from patients with chronic atrophic gastritis (CAG), intestinal metaplasia (IM), and gastric cancer (GC) were retrieved and integrated. Comparative analysis of the re-clustered epithelial cells identified 567 genes significantly upregulated in GC compared with IM, and 860 genes significantly upregulated in IM compared with CAG. The intersection of these two upregulated gene sets yielded 69 genes showing progressively increased expression along the gastritis → intestinal metaplasia → gastric cancer sequence, which may represent key molecular events driving malignant transformation of the gastric mucosa (Figure 6A,B). To further pinpoint targets involved in both disease progression and therapeutic response, we integrated these lesion-progression data with our transcriptomic drug intervention data. The intersection of the 69 progression-associated genes with the 89 berberine-responsive DEGs (model-upregulated and treatment-downregulated) yielded three shared genes: *S100A6*, *WFDC2*, and *DMBT1* (Figure 6C). These three genes are not only progressively upregulated along the CAG→IM→GC evolutionary spectrum, but also significantly upregulated in SPEM and downregulated after effective berberine treatment. Among these, *S100A6* exhibited the strongest evidence linking it to gastric carcinogenesis and immune response. At single-cell resolution, S100A6 upregulation was predominantly localized to malignant epithelial cells. In our SPEM model, S100A6 protein expression was significantly elevated in tamoxifen-treated mice and significantly decreased upon berberine treatment, as confirmed by both immunohistochemistry and immunofluorescence (Figure 6D,E). These observations suggested that S100A6 could play a vital role in berberine’s ability to alleviate SPEM and the malignant progression of precancerous lesions, and may hold potential value as an early warning marker for gastric cancer and a monitoring indicator for treatment response.

## 3. Discussion

In recent years, SPEM has been recognized as a key type of gastric metaplasia and has gained increasing attention for its role in the progression of gastric precancerous lesions [28,29]. The hallmark of SPEM is lineage reprogramming of chief cells in the gastric body, which shift from pepsinogen secretion to expressing molecular markers such as TFF2 and AQP5 [7,30]. Studies have shown that SPEM is not a static pathological phenomenon but a dynamic intermediate step involved in both gastric mucosal damage repair and malignant transformation: It serves as a reparative response initiated by acute injury (e.g., drugs or infection) yet may cross the boundary of normal repair under chronic persistent inflammation and evolve into the initiating stage of precancerous lesions [6]. Multiple clinical studies and animal experiments have further confirmed that SPEM, intestinal metaplasia (IM), and dysplasia exhibit substantial spatial overlap and a sequential evolutionary relationship, and are thus regarded as a key pathological step linking chronic gastritis and intestinal-type gastric cancer [31]. Therefore, effective intervention at this critical node—blocking or reversing SPEM progression—represents an important breakthrough for curbing gastric mucosal precancerous progression.

Previous studies have explored pharmacological interventions for SPEM. Xiong et al. demonstrated that Zuojin Capsule ameliorated SPEM by regulating multiple targets and pathways (e.g., PI3K-Akt, IL-17 signaling) [17]. Chen et al. reported that Yiwei Xiaoyu Granules promoted SPEM recovery via TFF2 and miR-7 regulation [32]. However, these drugs have limited efficacy. It is noteworthy that berberine—an inexpensive, orally bioavailable natural alkaloid—offers several potential advantages over these reported drugs: A well-characterized safety profile, established gastric mucosal bioavailability, and a multimodal mechanism that simultaneously addresses inflammation, metaplasia, and immune dysregulation. Modern pharmacological studies have confirmed that berberine induces autophagy in gastric cancer cells by inhibiting the MAPK/mTOR/p70S6K and Akt pathways [26], ameliorates atrophic gastritis by inhibiting IRF8/IFN-γ signaling [21], and reverses intestinal metaplasia and dysplasia by regulating cell cycle and apoptosis [23,24,25]. These findings suggest that berberine has the potential to intervene across the entire inflammation–metaplasia–carcinogenesis spectrum of gastric mucosa. We therefore focused this study on exploring the preventive and therapeutic effects of berberine on SPEM and its underlying mechanisms.

Through histopathological assessment and immunohistochemical/immunofluorescence staining of SPEM-specific markers (TFF2, AQP5, etc.), we found that berberine significantly reduced the severity of tamoxifen-induced SPEM in mice and promoted gastric mucosal recovery. Specifically, berberine pre-treatment partially prevented SPEM induction by attenuating parietal cell loss, while post-induction berberine administration significantly accelerated glandular restoration and SPEM resolution, advancing the recovery trajectory by approximately 3–4 days. These findings confirm that berberine exerts clear preventive and therapeutic effects on SPEM in vivo.

Recent studies have shown that the immune microenvironment—particularly M2 macrophage polarization driven by the IL-33-ST2-IL-13 signaling axis—plays a central role in SPEM pathogenesis: after acute gastric mucosal injury, subepithelial cells release the alarm signal IL-33, activating ILC2s and upregulating type 2 cytokines including IL-13, which exacerbates injury and induces M2 macrophage polarization and SPEM formation. M2 macrophages in turn secrete IL-33, further promoting SPEM development in a positive feedback loop [14,15]. Cell depletion studies have confirmed that macrophage depletion impairs SPEM development, whereas mice lacking other immune cells (T cells, B cells, neutrophils, eosinophils) still develop SPEM, underscoring the crucial role of M2 macrophages [14]. To explore the mechanism of berberine, we performed transcriptomic sequencing of mouse gastric mucosa. SPEM molecular markers such as *Wdfc2* were significantly upregulated after tamoxifen treatment and significantly downregulated after 3, 7, and 10 days of berberine treatment, consistent with previous reports [33]. Functional enrichment and KEGG analyses revealed that differentially expressed genes were significantly enriched in the JAK-STAT, NF-κB, and TNF signaling pathways. GSEA further confirmed that JAK-STAT and NF-κB signaling were significantly upregulated in the SPEM model and significantly downregulated after 3 days of berberine treatment, suggesting that berberine may promote SPEM recovery by inhibiting these pathways. The JAK-STAT pathway is closely linked to M2 macrophage polarization: the IL-4/IL-13-activated JAK1-STAT6 axis is the core pathway driving M2 polarization, upregulating M2-associated cytokines [34]. Additionally, NF-κB signaling exerts critical effects on M2 macrophage polarization. The p50 homodimer of NF-κB is a key transcriptional regulator orchestrating alternative M2 polarization. Upon IL-4 stimulation, the p50 homodimer can synergize with STAT6 to initiate transcription of M2 signature genes such as *Arg1*, which promotes M2 macrophage polarization [34,35,36]. Further transcriptomic analysis revealed that the M2 macrophage marker *CD163* was significantly upregulated in the model group and progressively downregulated at all berberine treatment time points; M2-associated genes such as *Lyve1* and *Cd9* showed similar trends [37,38,39,40]. Immunohistochemistry confirmed that the number and proportion of CD163^+^ M2 macrophages in the gastric mucosa significantly decreased after berberine treatment. Collectively, these findings indicate that berberine prevents and treats SPEM by regulating M2 macrophage polarization and infiltration through the JAK-STAT and NF-κB signaling pathways.

Our integrated single-cell analysis revealed *S100A6* as a progressively upregulated transcript across the gastritis–intestinal metaplasia–gastric cancer continuum. S100A6, a calcium-binding protein of the S100 family, has been implicated in multiple cancers, including gastric cancer. Mechanistically, it promotes the proliferation and migration of tumor cells by activating the MAPK pathway and upregulating key proteins of the cell cycle [41]. Clinically, high S100A6 expression is an independent prognostic factor for overall survival [42]. In the context of tumor immunity, S100 family proteins are well-established mediators of inflammation–cancer crosstalk: they can be secreted by activated macrophages and bind RAGE on immune cells, amplifying inflammatory signaling and macrophage recruitment [43,44]. Although the direct interaction between S100A6 and macrophages remains poorly defined, we observed that S100A6 upregulation in the SPEM model paralleled M2 macrophage infiltration, and berberine treatment downregulated S100A6 concomitant with reduced M2 macrophages and attenuated JAK-STAT/NF-κB pathway activity. These findings suggest that S100A6 may serve as a molecular bridge linking epithelial metaplasia and the immune microenvironment, and that berberine exerts its immunomodulatory effects partly by downregulating S100A6. Collectively, S100A6 emerges as a dynamic molecular linker connecting early gastric metaplastic lesions to malignant transformation, representing a promising biomarker for disease progression and a potential therapeutic target for anti-SPEM intervention.

This study has several limitations. First, our findings are derived from a single animal model (tamoxifen-induced SPEM). Validation in additional models, particularly Helicobacter pylori-induced SPEM, is needed. Second, all in vivo experiments were performed using a single berberine dose of 100 mg/kg, and dose–response relationships were not investigated in the current work. Therefore, we cannot determine the optimal effective concentration, the minimum therapeutic dosage, or the dose-dependent efficacy and safety range of berberine for SPEM intervention. Different drug concentrations may produce distinct phenotypic outcomes, immune regulatory effects, and molecular responses. Future studies incorporating multiple graded doses of berberine will be necessary to establish a more comprehensive dose–effect profile and further validate its therapeutic window for the prevention and reversal of gastric precancerous lesions. Third, although we demonstrate that berberine modulates M2 macrophages possibly via the JAK-STAT/NF-κB axis and S100A6, the precise molecular mechanisms and the causal relationship between S100A6 and macrophage polarization remain to be identified. Single-cell transcriptomic and proteomic approaches could provide higher-resolution mechanistic insights. Notably, berberine has poor oral bioavailability, implying that its therapeutic effects on gastric SPEM in our mouse model are predominantly mediated via local gastric mucosal action rather than systemic absorption [45,46]. In clinical practice, high-dose oral berberine may induce gastrointestinal adverse reactions, such as nausea, bloating, and diarrhea [47]. Therefore, rational dose conversion from animal data to human trials is required, and gastrointestinal tolerability needs to be closely monitored in future translational studies. Finally, all conclusions are limited to the mouse model; confirmation in human pathological specimens, patient-derived SPEM organoids, and large clinical cohorts is a critical next step.

## 4. Materials and Methods

### 4.1. Animal

Specific pathogen-free (SPF)-grade male C57BL/6 mice (18–22 g) were obtained from the Laboratory Animal Center of the Air Force Medical University (SYXK [Shaanxi] 2024-003) and housed under standard SPF conditions. All procedures were approved by the Institutional Animal Ethics Committee (approval number: 20250206).

### 4.2. Drugs and Reagents

Tamoxifen (T5648, Sigma-Aldrich, St. Louis, MO, USA), berberine (IC-0225779, InCellGene, Nanjing, China), methylcellulose (9004-67-5, MedChemExpress, Monmouth Junction, NJ, USA), mIHC kit–four-color TSA-Rab-275B (10289100020, Pengyue Biotechnology, Shenzhen, China), anti-TFF2 (1361-1-AP, Proteintech Group, Rosemont, IL, USA), anti-ATP4α (PA106346, Proteintech Group, Rosemont, IL, USA), anti-AQP5 (ab315855, Abcam, Cambridge, UK), anti-CD163 (ab182422, Abcam, Cambridge, UK), and anti-S100A6 (ab1134149, Abcam, Cambridge, UK) were used.

### 4.3. Animal Experimental Protocol

After one week of acclimatization, six-to-eight-week-old mice were randomly assigned to 3 groups using a random number table. Each group contained 6 mice (*n* = 6) at each experimental time point.

Model group: mice received tamoxifen (250 mg/kg, i.p.) for 3 consecutive days to induce SPEM. Controls received vehicle (10% ethanol/90% corn oil).

Prevention group: berberine (purity ≥98%, 100 mg/kg, p.o.) was administered daily for 10 consecutive days, beginning 7 days before and continuing throughout the 3-day tamoxifen induction period. Corresponding controls received berberine vehicle (0.5% methylcellulose) on the same schedule.

Treatment group: after the 3-day tamoxifen induction, berberine (100 mg/kg, p.o.) was administered daily for 3, 7, 10, or 14 consecutive days. Corresponding controls received berberine vehicle (0.5% methylcellulose) on the same schedule.

### 4.4. Sample Collection

Twenty-four hours after the last administration, mice were euthanized. The stomach was opened along the greater curvature; the forestomach and antrum were excised, and the corpus was divided longitudinally. One portion was fixed in 4% paraformaldehyde for paraffin embedding; the remainder was snap-frozen at −80 °C.

### 4.5. H&E Staining and Histology

Fresh gastric corpus tissues were fixed in 4% neutral paraformaldehyde for 24 h, dehydrated in a gradient alcohol series, cleared in xylene, infiltrated with paraffin wax, and embedded. Paraffin sections were stained with hematoxylin and eosin (H&E) and then observed and photographed under a light microscope.

### 4.6. Immunohistochemistry

Paraffin sections were deparaffinized and rehydrated, and antigen retrieval was performed by microwave heating in citrate buffer (pH 6.0). After blocking with blocking solution for 1 h at room temperature, the sections were incubated overnight at 4 °C with anti-TFF2 (1:500 dilution), anti-ATP4α (1:300 dilution), anti-AQP5 (1:500 dilution), and anti-CD163 (1:200 dilution). The next day, the sections were treated with 3% hydrogen peroxide for 20 min to block endogenous peroxidase activity, then incubated with the corresponding secondary antibodies for 1 h at room temperature. The sections were then stained with DAB chromogenic solution, counterstained with hematoxylin, dehydrated, and mounted. Stained sections were observed and photographed under a light microscope.

### 4.7. Immunofluorescence

Paraffin sections were deparaffinized and rehydrated, and antigen retrieval was performed by microwave heating in citrate buffer (pH 6.0). After blocking endogenous peroxidase with 3% hydrogen peroxide for 20 min and blocking with blocking solution for 1 h at room temperature, the sections were incubated with anti-TFF2 (1:500) overnight at 4 °C. The following day, sections were incubated with the corresponding secondary antibody for 30 min at room temperature and then with fluorescent detection reagent (1:200) for 30 min. Subsequently, a second round of antigen retrieval was performed in citrate buffer (pH 6), followed by blocking with 3% hydrogen peroxide for 20 min and blocking solution for 1 h. Sections were then incubated with anti-ATP4α (1:300) overnight at 4 °C. On the third day, sections were incubated with the corresponding secondary antibody for 30 min, then with fluorescent detection reagent (1:200) for 30 min. Nuclei were stained with DAPI (1:200) for 30 min at room temperature, and the sections were dehydrated and mounted. Immunofluorescence images were acquired with a fluorescence microscope.

### 4.8. Transcriptomic Analysis

Gastric total RNA was extracted using the MJZol total RNA extraction kit(Magen, Guangzhou, China). RNA integrity was assessed before library construction. Strand-specific mRNA libraries were prepared using the Illumina^®^ Stranded mRNA Prep Ligation kit (Illumina, San Diego, CA, USA), and transcriptomic sequencing was performed on the Illumina NovaSeq platform. Each experimental group contained 3 biological replicates (n = 3). Raw sequencing reads were subjected to quality control using FastQC and trimmed with Trimmomatic to remove low-quality reads and adaptor sequences. Clean reads were mapped to the *Mus musculus* reference genome (GRCm38) using HISAT2. Gene expression quantification was performed using featureCounts. Differentially expressed genes (DEGs) were identified using edgeR (v4.0.2; Padj ≤ 0.05, |fold change| ≥ 1.5). Key candidate genes were identified by Venn intersection of upregulated DEGs in the SPEM model group with downregulated DEGs at 3, 7, and 10 days of berberine treatment. Functional annotation included Gene Ontology (GO) enrichment, KEGG pathway analysis, and Gene Set Enrichment Analysis (GSEA) to comprehensively reveal the involved biological processes, molecular functions, and potential regulatory networks. The raw RNA-seq sequencing reads were deposited in the Sequence Read Archive (SRA) database under accession number PRJNA1530028.

### 4.9. Single-Cell Sequencing Analysis

Single-cell RNA-seq data integration. Two public human gastric single-cell RNA-seq datasets were retrieved from the Gene Expression Omnibus (GEO) database, with accession numbers GSM3954949 and GSM4546323. These datasets were generated from human gastric tissues and included samples with chronic atrophic gastritis (CAG), intestinal metaplasia (IM), and gastric cancer (GC). Samples representing these three pathological conditions were selected for integration analysis to explore the transcriptional landscape of gastric precancerous and cancerous lesions and complement our mouse SPEM findings.

Public scRNA-seq datasets covering chronic atrophic gastritis, intestinal metaplasia, and gastric cancer were obtained from GEO. Data were processed with the Seurat R package: quality control (filtering cells with outlier gene counts and excessive mitochondrial content), total-count normalization with log transformation, highly variable gene selection, principal component analysis (PCA), graph-based and K-means clustering, and t-SNE visualization. Cell-type annotation was performed via a two-step pipeline. Automatic annotation was first generated by SingleR using reference datasets from CellDex, followed by manual validation with canonical marker genes, including EPCAM, KRT8, and KRT18 for epithelial cells. Final cell identities were confirmed by combining SingleR predictions with manual marker assessment. Epithelial cell clusters were then subsetted for DEG analysis. Within epithelial cells, DEGs were calculated using the Wilcoxon rank-sum test in FindMarkers (default parameters). Genes detected in over 10% of cells in either group with |log_2_FC| > 0.25 were filtered, and the Benjamini–Hochberg correction was applied; genes with adjusted *p* < 0.05 were considered significant.

### 4.10. Statistical Analysis

Data are presented as mean ± SD. Two-group comparisons used two-tailed Student’s *t*-test; multiple-group comparisons used one-way ANOVA. Analyses were performed with GraphPad Prism 8.0. *p* < 0.05 was considered statistically significant.

## 5. Conclusions

This study demonstrates that berberine prevents and reverses gastric SPEM by inhibiting M2 macrophage polarization via regulating the JAK-STAT/NF-κB pathway and S100A6 expression. These findings provide preclinical evidence supporting further evaluation of berberine as a promising anti-SPEM agent, or even a chemopreventive agent for gastric cancer.

## Figures and Tables

**Figure 1:**
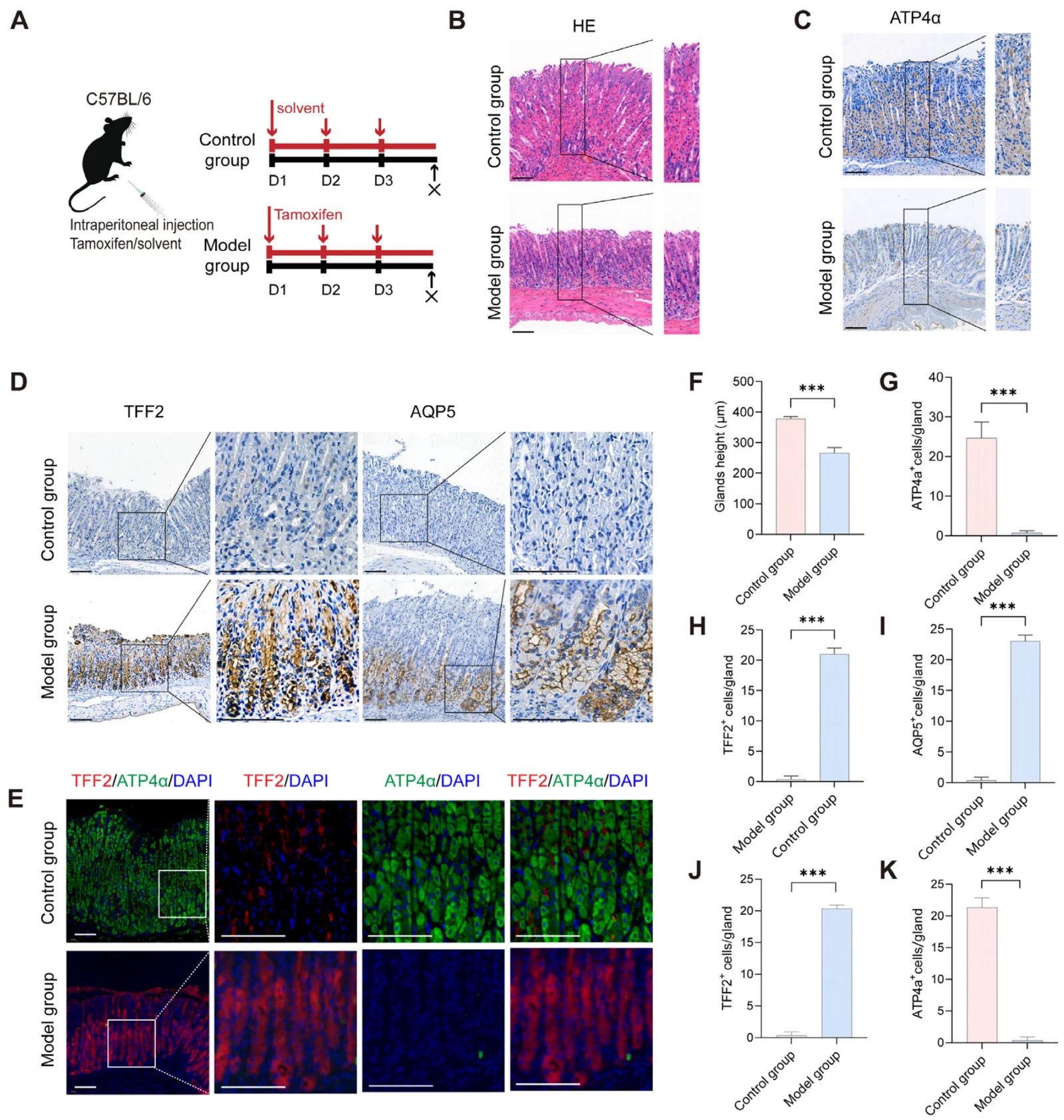
High-dose tamoxifen induces SPEM in mice. (**A**) Experimental protocol: tamoxifen (250 mg/kg/day, i.p.) or vehicle (10% ethanol + 90% corn oil) for 3 consecutive days. (**B**–**E**) H&E, IHC, and IF staining for ATP4α, TFF2, and AQP5 (Immunofluorescence staining for TFF2 (red), ATP4α (green), and DAPI (blue, nuclei)). (**F**–**K**) Quantification of gland height and positive cell counts. Scale bar, 100 μm. Data are mean ± SD; *** p < 0.001.

**Figure 2:**
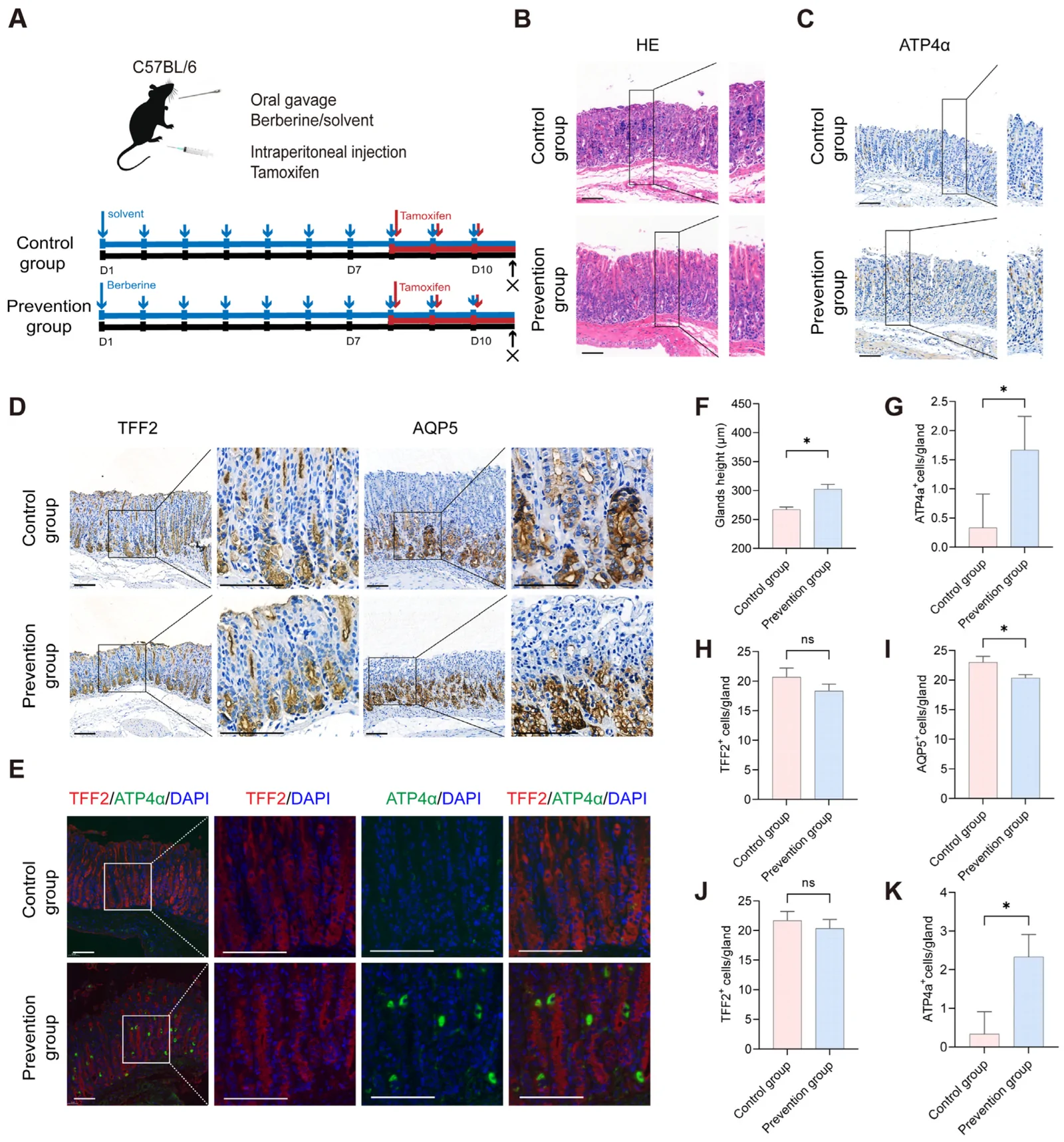
Berberine prevents SPEM development in mice. (**A**) Experimental protocol: berberine (100 mg/kg/day, p.o.) or vehicle for 10 consecutive days (7 days before + 3 days during tamoxifen induction). (**B**–**E**) H&E, IHC, and IF staining for ATP4α, TFF2, and AQP5. (Immunofluorescence staining for TFF2 (red), ATP4α (green), and DAPI (blue, nuclei)). (**F**–**K**) Quantification of gland height and positive cell counts. Scale bar, 100 μm. Data are mean ± SD; * *p* < 0.05; ns *p* ≥ 0.05.

**Figure 3:**
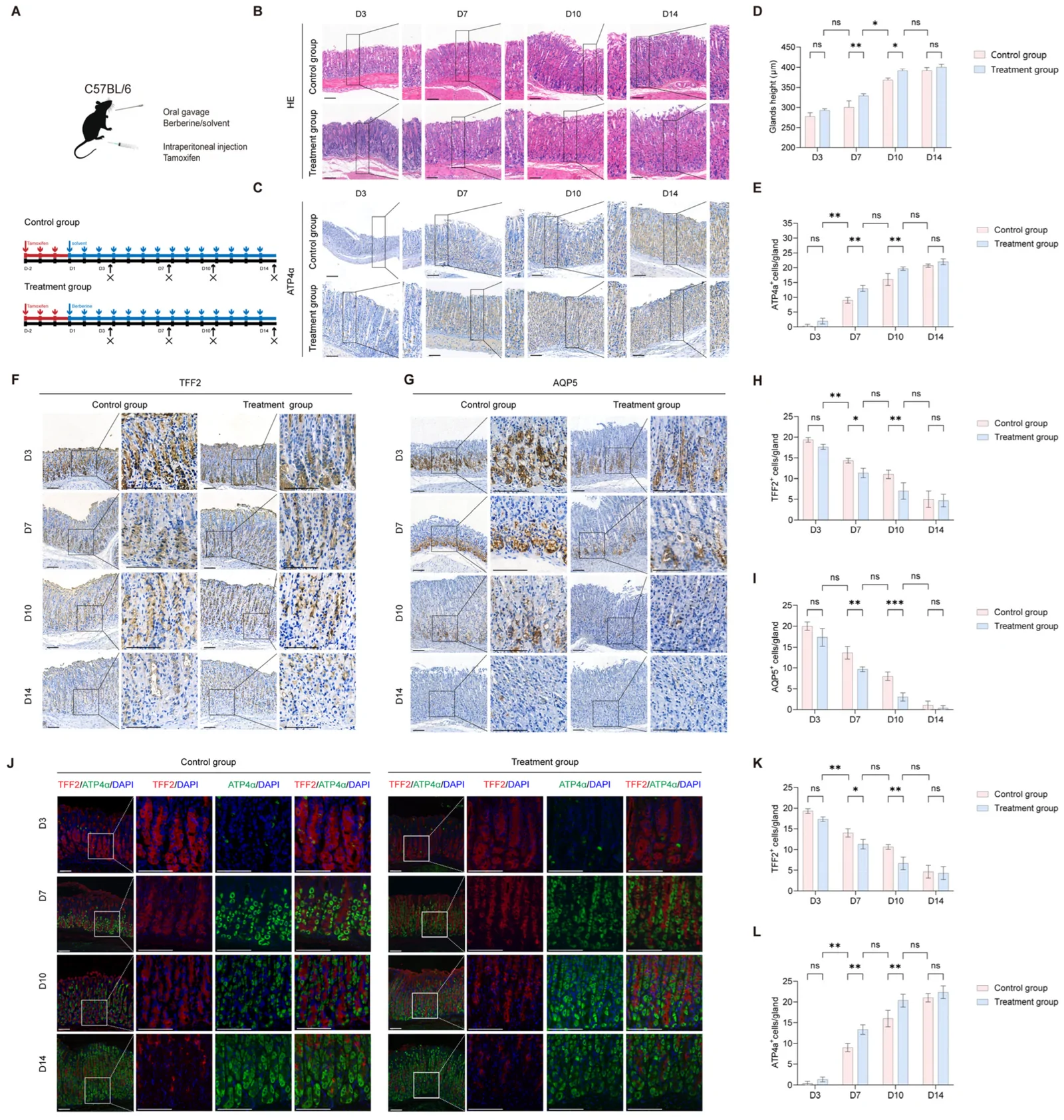
Berberine accelerates SPEM resolution in mice. (**A**) Experimental protocol: berberine (100 mg/kg/day, p.o.) or vehicle for 14 consecutive days after tamoxifen-induced SPEM; mice were sacrificed on days 3, 7, 10, and 14. (**B**,**C**,**F**,**G**,**J**) H&E, IHC, and IF staining for ATP4α, TFF2, and AQP5. (Immunofluorescence staining for TFF2 (red), ATP4α (green), and DAPI (blue, nuclei)). (**D**,**E**,**H**,**I**,**K**,**L**) Quantification of gland height and positive cell counts. Scale bar, 100 μm. Data are mean ± SD; * p < 0.05; ** p < 0.01; *** p < 0.001; ns *p* ≥ 0.05.

**Figure 4:**
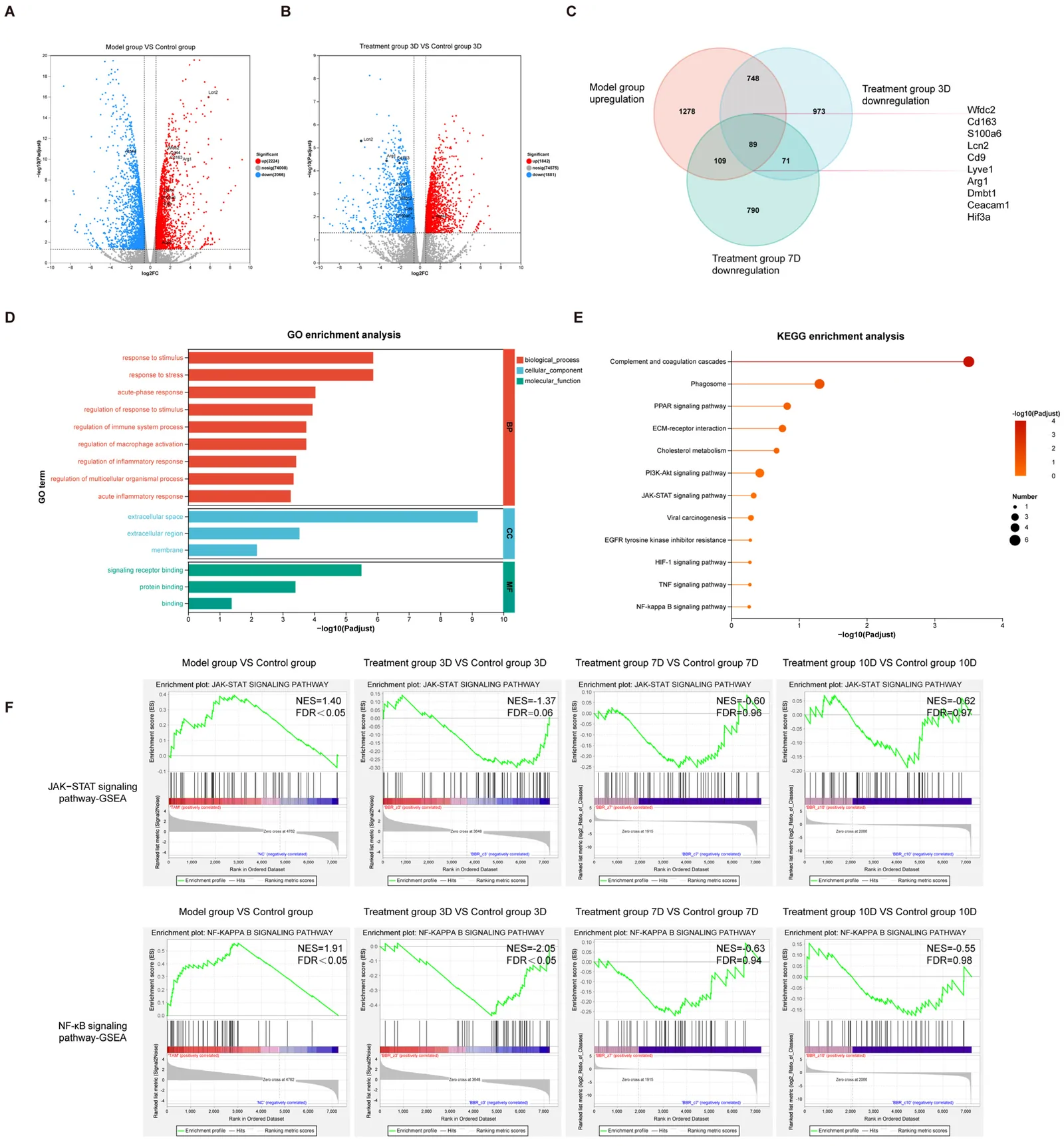
Transcriptomic profiling of berberine-treated gastric SPEM. (**A**,**B**) Volcano plots of DEGs in model vs. control and day 3 berberine vs. control. (**C**) Venn diagram of model-upregulated and berberine-downregulated genes. (**D**,**E**) GO and KEGG enrichment analyses of intersecting genes. (**F**) GSEA of NF-κB and JAK-STAT signaling pathways.

**Figure 5:**
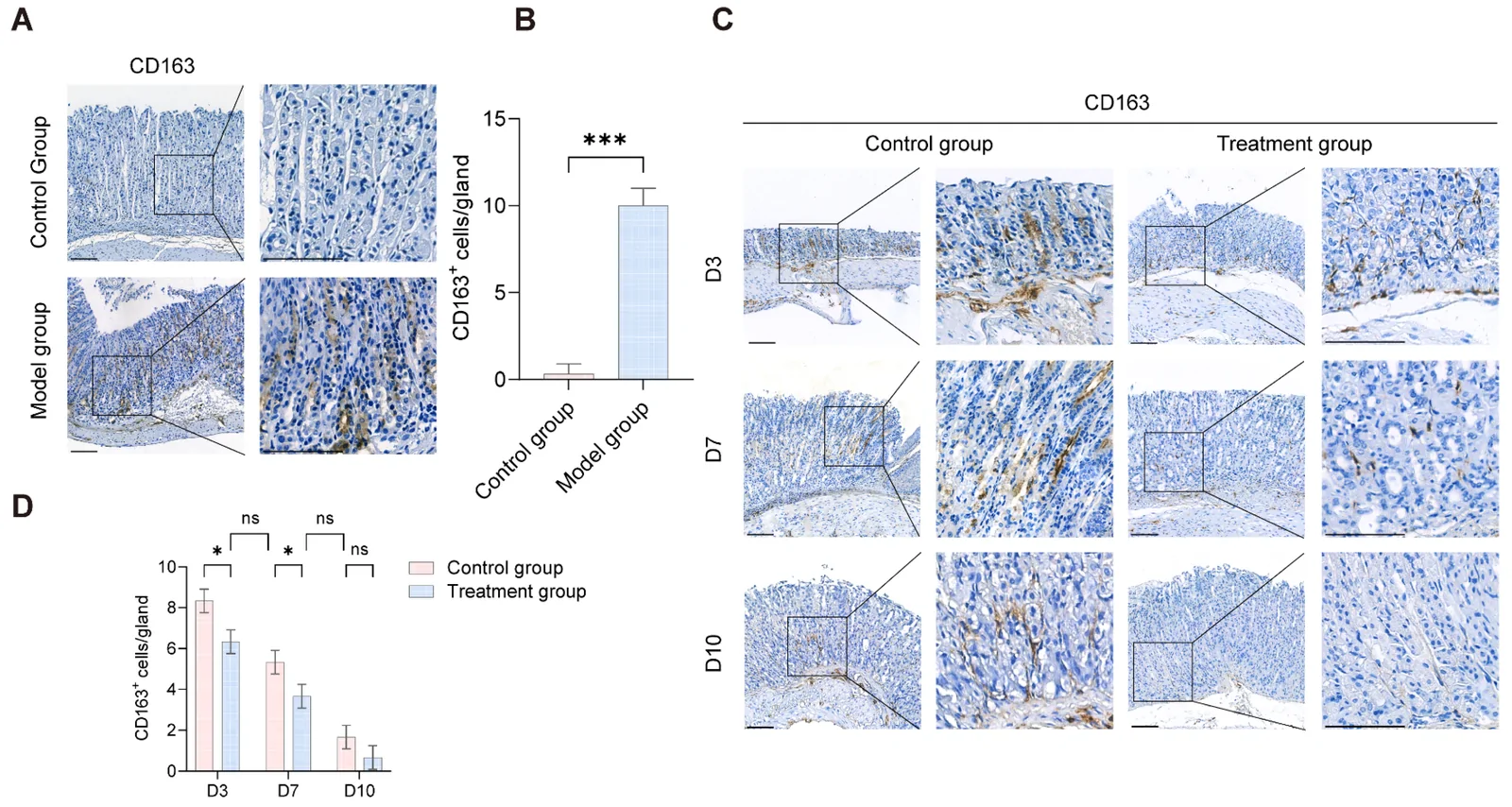
Berberine suppresses M2 macrophage polarization and infiltration in SPEM. (**A**,**C**) IHC staining for CD163 in model and treatment groups. (**B**,**D**) Quantification of CD163^+^ cell counts. Scale bar, 100 μm. Data are mean ± SD; * *p* < 0.05; *** p < 0.001; ns *p* ≥ 0.05.

**Figure 6:**
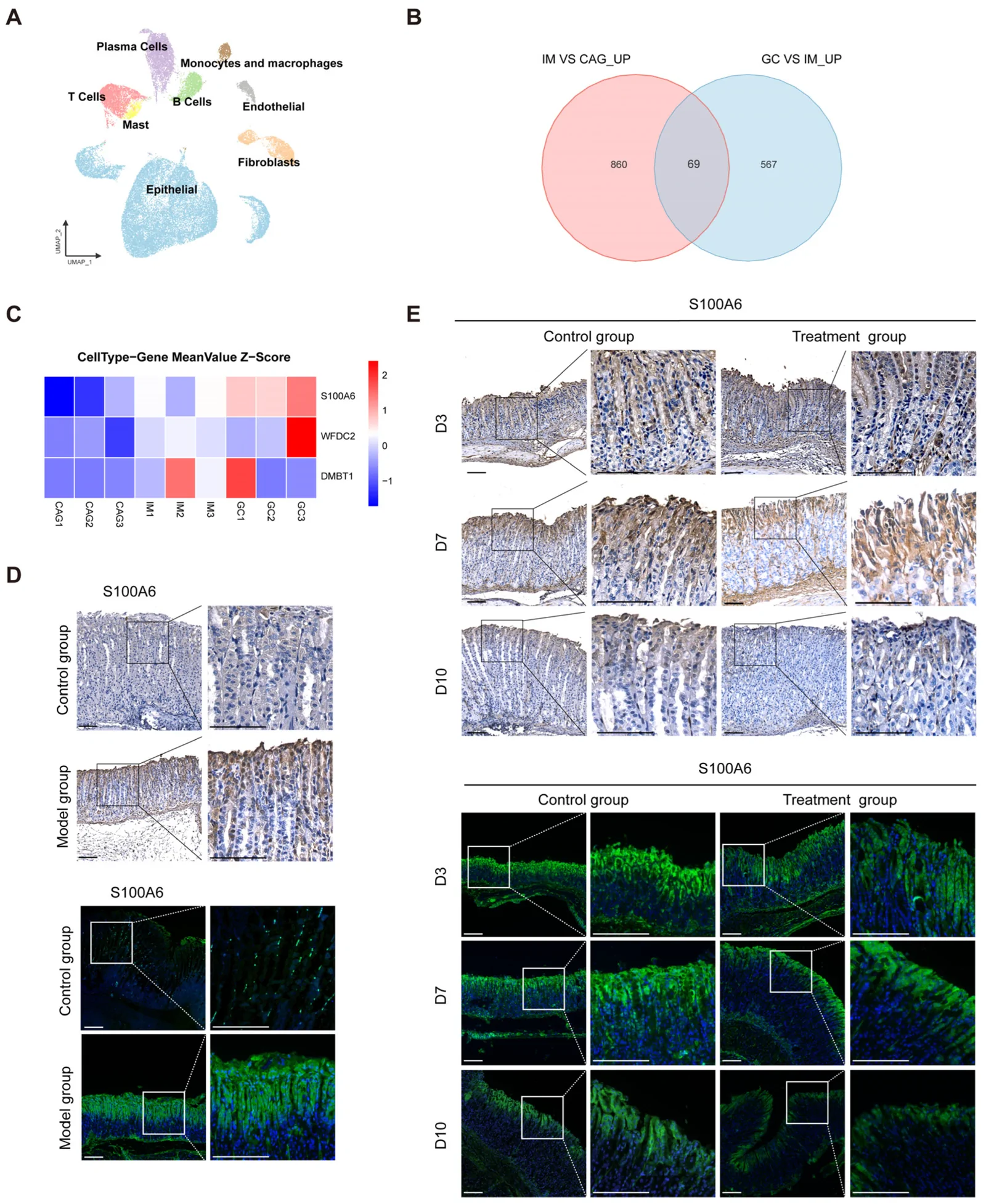
S100A6 as a candidate marker for CAG-IM-GC progression and treatment response. (**A**) Clustering analysis of scRNA-seq data from CAG, IM, and GC samples (epithelial, endothelial, B cells, T cells, etc.). (**B**) Venn diagram of upregulated DEGs in GC vs. IM and IM vs. CAG. (**C**) Heatmap of candidate genes including S100A6. (**D**,**E**) IHC and IF staining for S100A6 in gastric mucosa. (Immunofluorescence staining for S100A6 (green), and DAPI (blue, nuclei)). Scale bar, 100 μm.

## Data Availability

All the data generated or analyzed during this study are available from the corresponding author upon request. Raw sequencing data cannot be publicly deposited due to ongoing related follow-up studies. The processed datasets, original unprocessed imaging and experimental raw data that support the findings of this study are available from the corresponding author upon reasonable request.

## Abbreviations

The following abbreviations are used in this manuscript:

H&E	Hematoxylin and Eosin
IHC	Immunohistochemistry
IF	Immunofluorescence
pH	Potential of Hydrogen
µm	Micrometer
kg	Kilogram
mg	Milligram
i.p.	Intraperitoneal
p.o.	Per Os
FDR	False Discovery Rate
PCA	Principal Component Analysis
SPF	Specific Pathogen-Free
SRA	Sequence Read Archive
SPEM	Spasmolytic Polypeptide-Expressing Metaplasia
CAG	Chronic Atrophic Gastritis
IM	Intestinal Metaplasia
GC	Gastric Cancer
IL-33	Interleukin-33
IL-13	Interleukin-13
ST2	Interleukin-1 Receptor-Like 1
ILC2s	Group 2 Innate Lymphoid Cells
M2 macrophages	Alternatively Activated Macrophages
TFF2	Trefoil Factor 2
AQP5	Aquaporin 5
ATP4α	ATPase H^+^/K^+^-Transporting Subunit Alpha
CD163	CD163 Molecule
S100A6	S100 Calcium-Binding Protein A6
RNA	Ribonucleic Acid
DEGs	Differentially Expressed Genes
GO	Gene Ontology
KEGG	Kyoto Encyclopedia of Genes and Genomes
GSEA	Gene Set Enrichment Analysis
NF-κB	Nuclear Factor Kappa-Light-Chain-Enhancer of Activated B Cells
TNF	Tumor Necrosis Factor
JAK-STAT	Janus Kinase-Signal Transducer and Activator of Transcription
EPCAM	Epithelial Cell Adhesion Molecule
KRT8	Keratin 8
KRT18	Keratin 18
